# Effect of Preparation Methods on the Tensile, Morphology and Solar Energy Conversion Efficiency of RGO/PMMA Nanocomposites

**DOI:** 10.3390/polym9060230

**Published:** 2017-06-18

**Authors:** Shin Yiing Kee, Yamuna Munusamy, Kok Seng Ong, Koon Chun Lai

**Affiliations:** 1 Department of PetroChemical Engineering, Faculty of Engineering and Green Technology, Universiti Tunku Abdul Rahman, Jalan Universiti, Bandar Barat, Kampar, Perak 31900, Malaysia; nicolekee88@hotmail.com (S.Y.K.); laikc@utar.edu.my (K.C.L.); 2 Department of Industrial Engineering, Faculty of Engineering and Green Technology, Universiti Tunku Abdul Rahman, Jalan Universiti, Bandar Barat, Kampar, Perak 31900, Malaysia; skong@utar.edu.my

**Keywords:** RGO, PMMA, solar energy conversion efficiency, morphology, synthesis method

## Abstract

In this study, reduced graphene oxide (RGO)/polymethyl methacrylate (PMMA) nanocomposites were prepared by employing in situ polymerization and solution blending methods. In terms of mechanical properties, RGO loading increased the Young’s modulus but decreased the elongation at break for RGO/PMMA nanocomposites. Tensile strength for solution blended RGO/PMMA nanocomposites increased after adding 0.5 wt % RGO, which was attributed to the good dispersion of RGO in the nanocomposites as evidenced from SEM and TEM. Solar energy conversion efficiency measurement results showed that the optimum concentration of RGO in the RGO/PMMA nanocomposites was found to be 1.0 wt % in order to achieve the maximum solar energy conversion efficiency of 25%. In the present study, the solution blended nanocomposites exhibited better overall properties than in situ polymerized nanocomposites owing to the better dispersion of RGO in solution blending. These findings would contribute to future work in search of higher conversion efficiency using nanocomposites.

## 1. Introduction

Graphene is a monolayer hexagonal sp^2^ hybridized carbon sheet. It has received much attention in recent years due to its high mechanical strength, large specific surface area, and excellent thermal and electrical conductivity [1,2]. Graphene can be synthesized through various methods, such as chemical vapor deposition (CVD), plasma enhanced CVD (PECVD), graphitization of a carbon-containing substrate, solvothermal, organic synthesis, and chemical reduction of graphene oxide [3,4,5]. Graphene obtained by the reduction of graphene oxide through thermal, chemical or electrical treatments is generally known as reduced graphene oxide (RGO). Research works involving graphene/polymer nanocomposites have been carried out widely with regard to many applications, such as electromagnetic shielding, corrosion-resistant coating, antistatic, lithium ion batteries, supercapacitors, fuel cells, photovoltaic devices, biosensing systems and photocatalysts [6,7,8]. Researchers have synthesized graphene/polymer nanocomposites with several polymer matrices such as polystyrene, poly(vinyl alcohol), low density polyethylene, polymethyl methacrylate, epoxy and natural rubber composite using different preparation methods like melt intercalation, solution blending, in situ polymerization, etc. [9,10,11,12,13,14,15,16,17]. Despite various materials having been proposed for heat dissipation [18,19], the highly thermally conductive graphene-based nanocomposites have recently been the subject of focus in thermal management applications. Previous works showed that graphene can significantly improve the electrical, thermal and mechanical properties of graphene/polymer composites. Pham et al. [20] reported that the electrical conductivity of PMMA-RGO composites prepared by a simple latex emulsion polymerization method was 64.1 S/m at 2.7 vol % of RGO. Additionally, the thermal conductivity of epoxy and a mixture of graphene and multilayer graphene at 10 vol % loading was found to be about 2400% higher than the thermal conductivity of pristine epoxy [21].

Graphene is efficient for energy harvesting, which is attributed to its large optical absorptivity and charge mobilities. Optical absorption in graphene is dominated by intraband transitions at low photon energies (in the far-infrared spectral range) and interband transitions at higher energies (from mid-infrared to ultraviolet) [22]. During the interband transitions, the phonons (lattice vibration quanta) are exchanged with the lattice, and the reactions lead to a high thermal capacity. The absorption peak for PMMA and RGO is at about 190–260 nm and 268 nm, respectively [23,24]. The solar energy conversion efficiency of graphene/polymer nanocomposites is important for extending the application of graphene into thermal energy storage [25,26,27]. Though many research works have been carried out on the measurement of thermal conductivity and electrical conductivity of graphene/polymer nanocomposites, solar energy conversion efficiency of the graphene/polymer nanocomposites has nevertheless not gained much attention. Thus, the effect of PMMA/RGO preparation methods on the tensile strength, morphological properties and solar energy conversion efficiency of the nanocomposites should be studied in detail. PMMA is selected in this study, owing to its optically clear amorphous thermoplastic behavior, with high light transmittance and its resistance to chemical and weathering effects [28]. As the tensile properties of the RGO/PMMA nanocomposites depend strongly on the dispersion quality and interfacial interaction between the graphene and polymer matrix, the selection of graphene/polymer nanocomposites preparation methods needs to be taken into serious consideration. In this work, two preparation methods, namely in situ polymerization and solution blending are proposed to synthesize the RGO/PMMA nanocomposites. The effect of these two preparation methods on the morphology and mechanical properties of the RGO/PMMA nanocomposites will be reported and discussed. Moreover, the optimum concentration of RGO/PMMA nanocomposites for achieving the highest solar energy conversion efficiency will be investigated.

## 2. Materials and Methods

### 2.1. Raw Materials

Graphite nanofiber (GNF) was supplied by Platinum Sdn. Bhd, Senawang, Malaysia. Whereas Methyl methacrylate (MMA, 99%) was acquired from Merck, Selangor, Malaysia. Polymethyl methacrylate, PMMA (120,000 molecular weight) and hydrazine hydrate solution (78%–82% iodometric) were purchased from Sigma Aldrich, Subang Jaya, Malaysia. Sulfuric acid (95%), hydrogen peroxide (30%), hydrochloric acid (37%), chloroform (stabilized with 0.6%–1.0% ethanol) and azobisisobutyronitrile (AIBN) were purchased from R&M Chemicals, Semenyih, Malaysia. Potassium permanganate and sodium nitrate were obtained from Bendosen Laboratory Chemicals, Johor Bahru, Malaysia. Deionized water was used throughout oxidation, reduction and polymerization process.

### 2.2. Preparation of Graphene Oxide (GO)

Graphene oxide (GO) was prepared following the conventional Hummer’s method [29]. 5 g of GNF and 2.5 g of sodium nitrate were added into a beaker filled with 115 mL of sulfuric acid. After the sodium nitrate dissolved, 15 g of potassium permanganate was slowly added followed by 230 mL of deionized water. The solution was then stirred for 3 h before being poured into 700 mL of deionized water and mixed with 12 mL of hydrogen peroxide. The mixture was then left overnight and filtered using Anodisc membrane. The filtrate cake was washed with 5% hydrochloric acid and then with deionized water repeatedly until the pH of the supernatant reached 5–7. Finally, the product was dispersed in deionized water and dried overnight in an oven at 60 °C.

### 2.3. Preparation of Reduced Graphene Oxide (RGO)

500 mg of GO was dispersed in 500 mL of deionized water and then exfoliated in an ultrasonicator bath for 1 h. Subsequently, 5 mL of hydrazine hydrate was added and the solution was refluxed for 24 h. The solution turned from brown to black precipitate. The product was filtered, washed with deionized water and dried at 60 °C in vacuum oven.

### 2.4. Preparation of RGO-PMMA Composite

#### 2.4.1. In Situ Polymerization of RGO-PMMA Nanocomposite

RGO was dispersed in MMA in a glass reactor and sonicated for 1 h, followed by the adding of 0.5 wt % initiator AIBN. The reaction mixture was then heated in a water bath at 70 °C for 4 h and in an oven at 100 °C overnight for the completion of polymerization.

#### 2.4.2. Solution Blending of RGO-PMMA Nanocomposite

RGO and PMMA were stirred separately in chloroform. Subsequently, the RGO/chloroform solution was added to the PMMA/chloroform solution and stirred for 1 h. The solution was then poured into the Teflon mould. Finally, the chloroform was evaporated in vacuum oven at 50 °C.

### 2.5. Characterization of Nanofillers and Nanocomposites

Raman spectra of GNF and GO were recorded using NT-MDT NTEGRA Spectra instrument in order to observe the structural information of the samples. The excitation wavelength was 473 nm and the laser power was set to 1.7 mW. All powder samples were directly deposited on the glass slide in the absence of solvents. The XPS data were taken using ULVAC-PHI Quantera II equipped with monochromatic Al Kα X-ray source (hν = 1486.6 eV). All spectra were collected at room temperature. The ratio of C to O atomic element composition for GNF, GO and RGO was measured by calculating the peak areas of C and O elements from the spectra. Additionally, the infrared spectra of GNF, GO, RGO and RGO-PMMA nanocomposites were recorded using Nicolet photo-spectrometer 8700 to obtain the information about functional groups in the sample. The KBr pellet was used to analyze the absorption band with a wavelength range from 4000 to 400 cm^−1^ with 32 scans.

Tensile testing was carried out in accordance with the standard test method ASTM D882-10 by employing Tinius Olsen H10KS lightweight tensile tester with a crosshead speed of 5 mm/min. The morphologies of GNF, GO, RGO and fracture surfaces of RGO/PMMA nanocomposites at different magnifications were captured by JEOL JSM-6701F scanning electron microscope (SEM, JEOL Inc., Massachusetts, MA, USA). Each sample was examined after sputter coating with a thin layer of platinum to avoid electrostatic charging and poor image resolution. A Hitachi HT7700 transmission electron microscope (TEM) was employed to acquire the images of RGO, RGO/PMMA nanocomposites at an accelerating voltage of 100 KV. Samples were sectioned in epoxy resin embedment before being viewed using TEM.

### 2.6. Solar Energy Conversion Efficiency

The specific heat capacity of every nanocomposite sample was measured using Mettler Toledo TOPEM differential scanning calorimeter (DSC, Mettler Toledo Inc., Greifensee, Switzerland) prior to measuring their solar energy conversion efficiencies. The solar energy conversion efficiency measurement was carried out under outdoor conditions.

#### 2.6.1. Specific Heat Capacity Measurement

The specific heat capacity of nanocomposite samples from the DSC result can be calculated by:

(1) $$c_{s}=\frac{\Delta Q}{m_{s}\Delta T}=\frac{\frac{\Delta Q}{\Delta t}}{m_{s}\left(\frac{\Delta T}{\Delta t}\right)}$$

where *c_s_* and *m_s_* are specific heat capacity of the nanocomposite sample (Jg^−1^·K^−1^) and mass of the sample (g), respectively. Δ*Q* is heat energy supplied to the nanocomposite sample (J), Δ*T* is the change in temperature (K) and Δ*t* is the period of time for the change in temperature Δ*T* (s).

#### 2.6.2. Outdoor Solar Energy Conversion Efficiency Measurement

**Figure 1:**
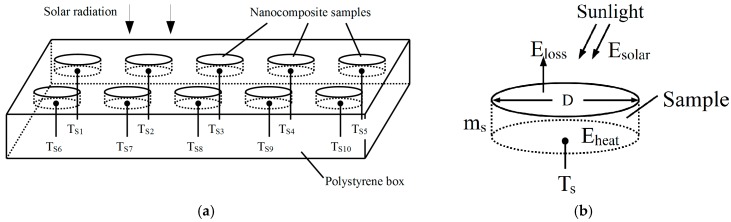
(**a**) Experiment setup for solar energy conversion system testing; (**b**) Heat transfer of a nanocomposites sample.

Samples of RGO/PMMA nanocomposites were placed in the polystyrene box and exposed to the sun as illustrated in Figure 1.

**Figure 2:**
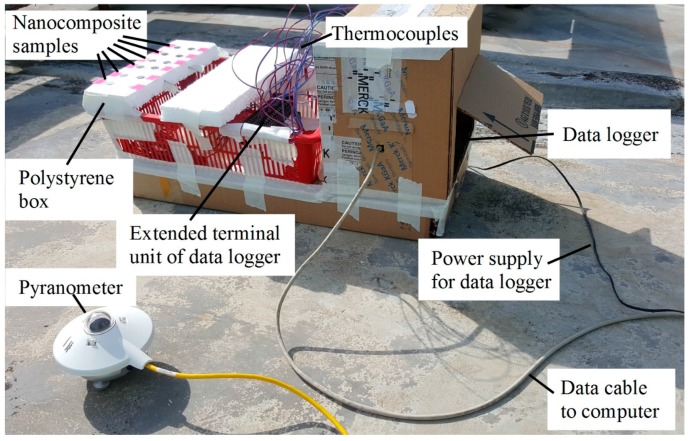
Outdoor experiment setup for solar energy conversion efficiency measurement.

Figure 2 illustrates the outdoor experimental setup for solar energy conversion efficiency measurement. A KiPP & Zonen CMP3 Pyranometer was used to measure the solar irradiance in Watts per meter square. Temperatures of the nanocomposite samples (denoted as T_s1_–T_s10_) were recorded using thermocouples connected to Graphtec data logger GL820, before being processed by a computer.

Heat transfer for a nanocomposite is shown in Figure 1b. Solar energy (*E_solar_*) is generally absorbed at the top surface of the sample. A portion of the solar energy is converted into heat energy (*E_heat_*), which substantially increases the temperature of the sample (*T_s_*). On the other hand, sunlight reflected from the top surface and radiative heat transfer to the environment account for heat loss (*E_loss_*) from the sample. Therefore, the rate of energy absorbed by the sample is represented by:

(2) $$E_{heat}=E_{solar}-E_{loss}=m_{s}c_{s}\Delta T_{s}$$

The total solar energy incident on the top surface area of the sample over a period of time is given by:

(3) $$E_{solar}=\int_{t=0}^{t_{N}}I_{solar}\left(t\right)\cdot A\;dt$$

where *I_solar_* is the solar irradiance measured by using the pyranometer (Wm^−2^) and *A* (π*D^2^/4) is the top surface area of the nanocomposite sample (m^2^).

Finally, the solar energy conversion efficiency (*η*) can be thus determined as:

(4) $$\eta =\frac{E_{heat}}{E_{solar}}\times 100\%$$

## 3. Results and Discussion

### 3.1. Characterization of the Nanofiller and Nanocomposites

Evaluations using FTIR, Raman spectroscopy and XPS were carried out to determine the oxidation and reduction of GNF, and to substantially characterize the chemical compositions and morphology of GNF, GO, RGO and RGO/PMMA nanocomposites.

#### 3.1.1. Raman Spectroscopy

**Figure 3:**
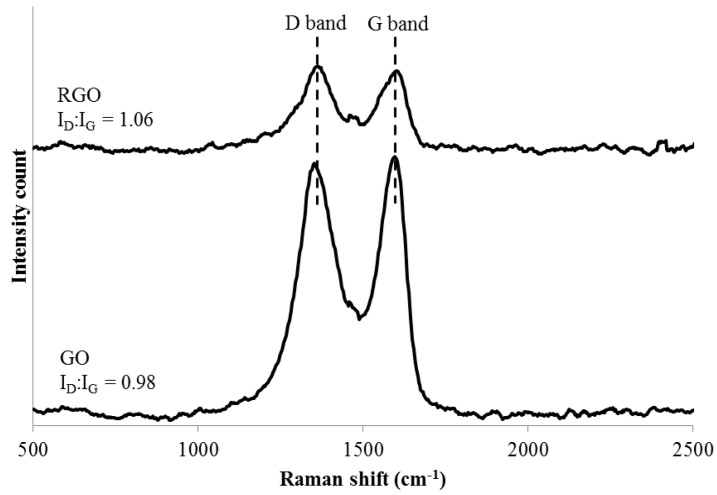
Raman spectra of GO and RGO.

Strong G and D peaks can be seen from the Raman spectra in Figure 3 for both GO and RGO, suggesting that they have very small crystal sizes [30]. According to the Raman spectrum of GO, the G and D bands are at 1597 and 1353 cm^−1^, respectively. The G band corresponds to the first-order scattering of the E^2^g mode, whereas the prominent D peak indicates the reduction in size of the in-plane sp^2^ domain. This is due to the extensive oxidation and the attachment of hydroxyl and epoxide groups on the carbon basal plane. On the other hand, the Raman spectrum of RGO indicates the G band at 1603 cm^−1^ and D band at 1363 cm^−1^. Since the D band of RGO is more intense than the G band, it implies a reduction of the exfoliated GO results in a decrement of the average size of sp^2^ domain [31].

#### 3.1.2. X-ray Photoelectron Spectroscopy (XPS)

**Figure 4:**
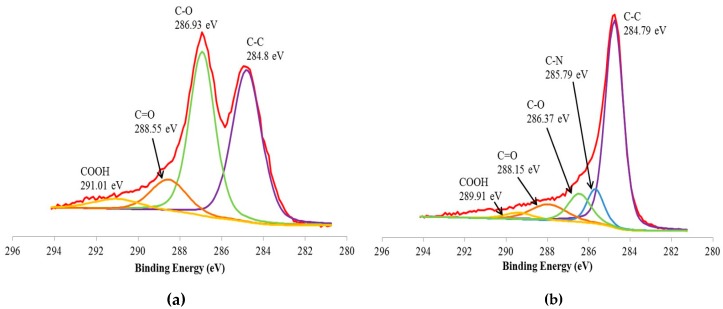
The C1s XPS spectrum of (**a**) GO; (**b**) RGO.

Figure 4 shows the core-level shift (Cls) XPS spectra of GO and RGO, respectively. It is apparent from Figure 4a that the GNF was oxidized to GO. The Cls XPS spectrum of GO are represented by the characteristic peaks corresponding to carbon atom with different functional groups: the non-oxygenated ring C (C–C, 284.80 eV), ether C (C–O, 286.93 eV), carbonyl C (C=O, 288.55 eV) and carboxylate C (COOH, 291.01 eV). The Cls spectrum of RGO in Figure 4b exhibits the similar oxygen functionalities, however, their peak intensities are much smaller than those in GO, possibly owing to the reduced oxygen functional groups. Besides, an additional peak at 285.79 eV corresponding to nitrogen bound to carbon atom was noticed [32]. This indicates that the reduction of GO to RGO using hydrazine hydrate will incorporate nitrogen to the carbon atom [33].

#### 3.1.3. Fourier Transform Infrared Spectroscopy (FTIR)

**Figure 5:**
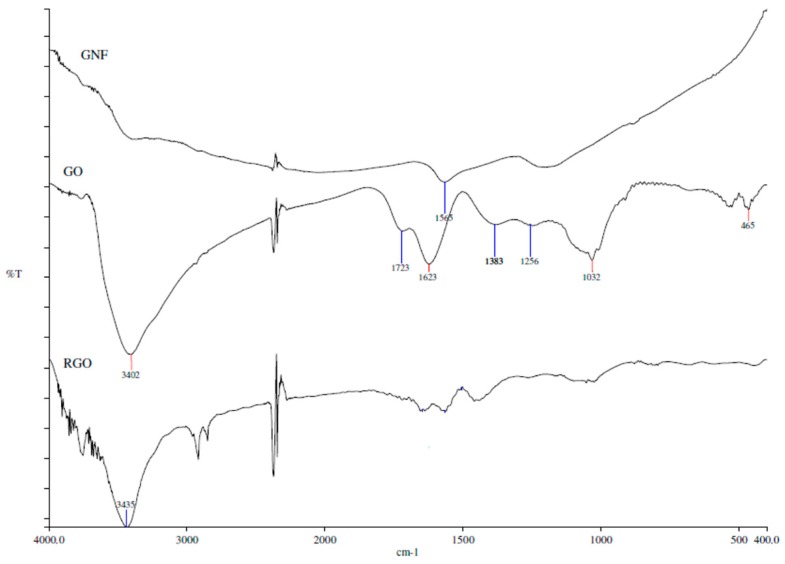
FTIR spectra of GNF, GO and RGO.

Figure 5 depicts the FTIR spectra of GNF, GO and RGO. The peak of GNF at 1565 cm^−1^ corresponds to the C=C aromatic ring chain stretching. The spectrum of GO represents the characteristic peaks corresponding to the oxygen functionalities group such as C=O stretching, C–O stretching and C–O bending vibration at 1723, 1256 and 1032 cm^−1^, respectively [34]. The peak at 3402 and 1386 cm^−1^ corresponds to hydroxyl groups (OH), whereby the broad range of 2500 to 3500 cm^−1^ confirms the presence of carboxylic group (COOH). Moreover, the peak at 1623 cm^−1^ represents the unoxidized graphitic domain (C=C). The GO spectra show that the oxidation of GNF using Hummer’s method was successful through the incorporation of the oxygen functional group. The reduction of GO was confirmed by the FTIR spectrum of RGO in that the peak intensity of all oxygen functionality groups was reduced significantly. Note that the peak at 3435 cm^−1^ (represents OH) in the FTIR spectrum of RGO indicates the reduction of GO was incomplete.

**Figure 6:**
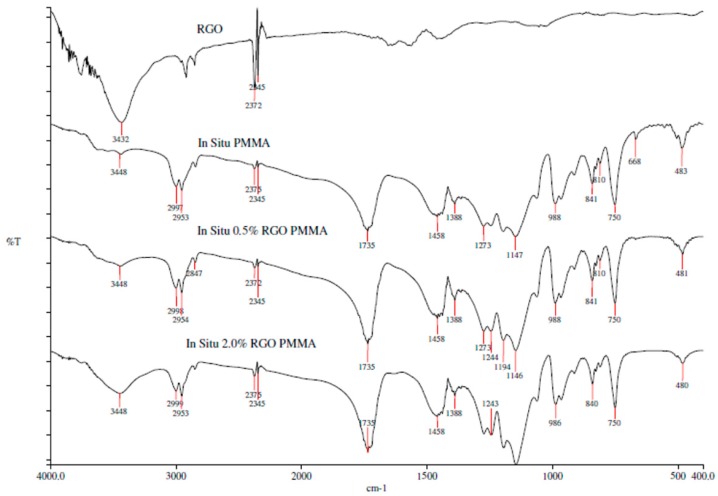
FTIR spectra of RGO, PMMA and nanocomposites prepared by in situ polymerization.

**Figure 7:**
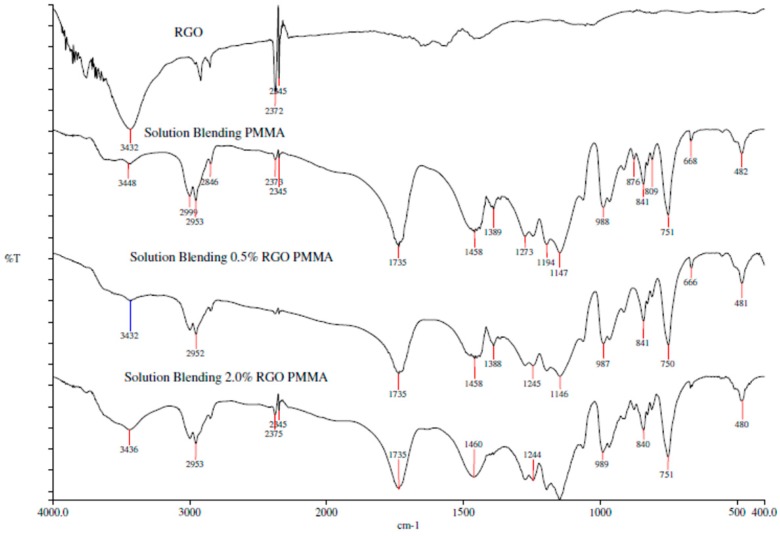
FTIR spectra of RGO, PMMA and nanocomposites prepared by solution blending.

The FTIR spectra of RGO, PMMA and the nanocomposites prepared by in situ polymerization and solution blending are shown in Figure 6 and Figure 7, respectively. As can be seen, 2.0% RGO PMMA nanocomposites have a broader and more intense peak at 3448 cm^−1^ compared to the neat PMMA. This may be due to the presence of the hydroxyl group in the RGO. In addition, interaction of PMMA and RGO may not involve chemical reaction, as no new functional group was obviously noticed under these preparation methods.

### 3.2. Mechanical Properties

The effect of the preparation method and RGO loading on the tensile properties of the nanocomposites are summarized in Table 1. It is apparent that the neat PMMA prepared via in situ polymerization exhibits relatively better performance than solution blending. In particular, for in situ nanocomposites, the Young’s modulus achieves its peak at 0.5 wt % RGO loading, but is at its minimal value at 2.0 wt % loading within the design window. Likewise, reduction of tensile strength can be seen. This may be caused by formation of an intercalated structure of RGO in the polymer matrix. The monomers polymerize between the RGO sheets, only expanding the distance between the sheets without fully destroying the ordered structure. In other words, an inhomogeneous dispersion of RGO will be achieved only if few monomers manage to get in between the sheets. This will reduce the surface area for interaction between the RGO and PMMA, and subsequently decrease the stress transfer efficiency.

With regard to solution blended PMMA nanocomposites, on the other hand, the addition of RGO up to 2.0 wt % leads to improvement of Young’s modulus, up to 46.79% compared to PMMA without RGO. The tensile strength with 0.5 wt % loading of RGO leads to slight improvement of 3.03%. However, addition beyond 0.5 wt % decreases the tensile strength, which may be attributed to the RGO agglomeration effect and poor stress transfer [35]. The elongation at break decreases with increasing RGO loading for nanocomposites prepared by both solution blending and in situ polymerization methods. The decrease in elongation at break may be attributed to the demobilization of polymer chains on the surface of RGO, which reduces the flexibility of the chain when the RGO loading increases. The decrease in elongation at break is more prominent for nanocomposites prepared by in situ polymerization due to inhomogeneous dispersion of RGO.

**Figure 8:**
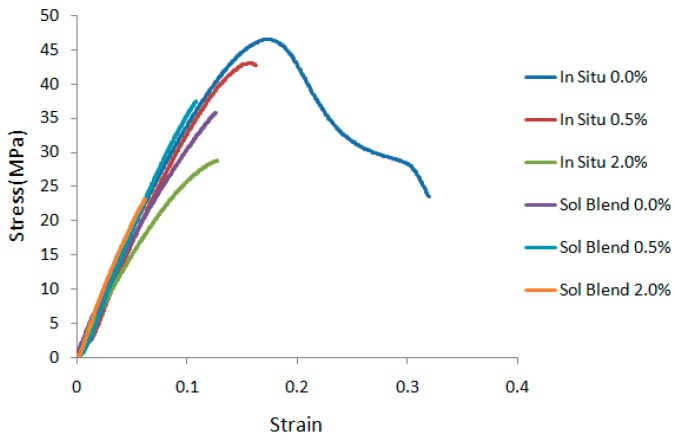
Stress-strain curves for various nanocomposites prepared by in situ polymerization and solution blending method.

To better illustrate the mechanical behavior, the stress-strain curves of the nanocomposites are shown in Figure 8. In the figure, a ductile behavior can be seen for the neat PMMA prepared by in situ polymerization method. However, all other samples displayed brittle fracture behavior. For nanocomposites prepared by the in situ polymerization method, the stress at break decreased with increasing RGO loading. Whereas, for those prepared by solution blending method, the stress at break was increased by adding 0.5% RGO, but decreased with further RGO loadings.

### 3.3. Morphology Properties

#### 3.3.1. Scanning Electron Microscope (SEM)

**Figure 9:**
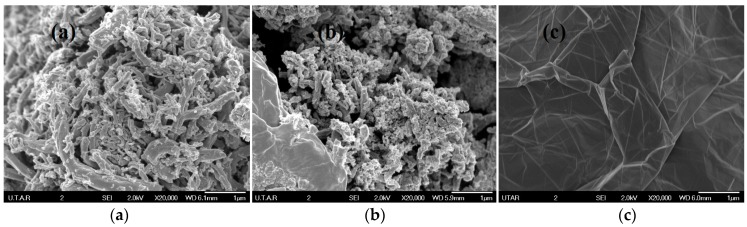
SEM images of (**a**) GNF; (**b**) GO; (**c**) RGO.

Figure 9 displays the FESEM images of GNF, GO and RGO morphology at 20,000 times magnification. Both GNF and GO possessed hair-like fiber structures; however, the GO fiber structure was torn into smaller pieces and the surface was rougher, when compared with GNF. This could be attributed to the oxygen-containing groups present on the surface of the graphitic layer.

**Figure 10:**
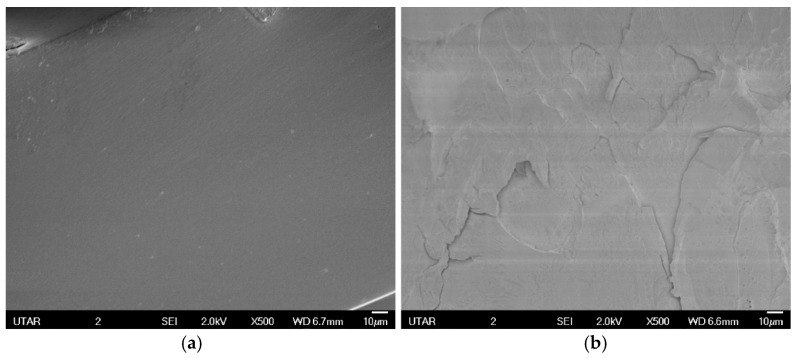
Tensile fracture surfaces of neat PMMA prepared by different methods. (**a**) In situ polymerization; (**b**) solution blending.

The cross-section morphology of the fractured nanocomposites was recorded by SEM at the magnification of ×500. The tensile fracture surfaces of the neat PMMA prepared by both methods are distinct from each other, as shown in Figure 10. Neat PMMA through in situ polymerization has a relatively smooth surface, whereas those prepared through solution blending have prominent crack growth on a major plane.

**Figure 11:**
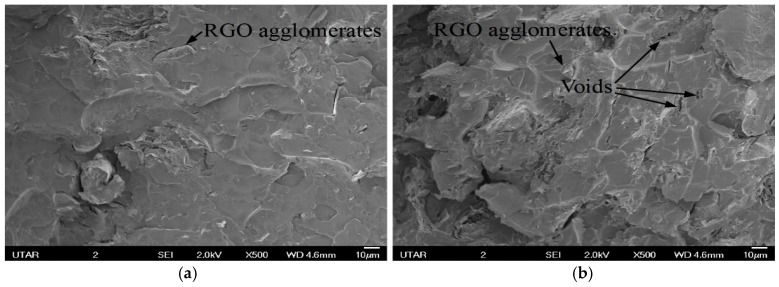
Tensile fracture surfaces of nanocomposites prepared by in situ polymerization with (**a**) 0.5 wt % RGO loading; (**b**) 2.0 wt % RGO loading.

**Figure 12:**
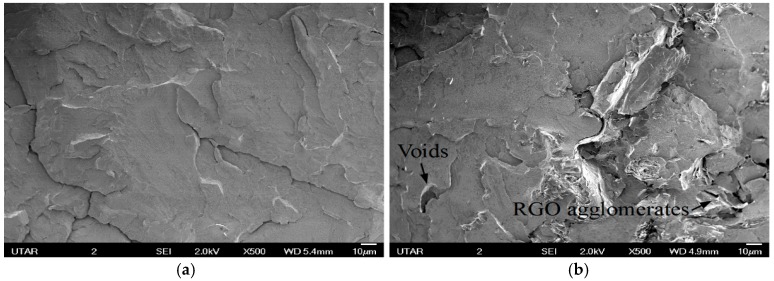
Tensile fracture surfaces of nanocomposites prepared by solution blending with different RGO loading. (**a**) 0.5 wt % RGO loading; (**b**) 2.0 wt % RGO loading.

Tensile strength for PMMA prepared by in situ polymerization was decreased by adding RGO. According to Figure 11, voids and RGO agglomerates can be observed on the tensile fracture surface of nanocomposites. Formations of agglomerate reduce the surface area of RGO for interaction with the polymer matrix and lead to poor adhesion between filer and matrix [36]. Voids can be attributed to the easy detachment of these agglomerates from the matrix [37]. These agglomerates also become stress concentration points in the nanocomposite system. Figure 12 shows the tensile strength for solution blended PMMA nanocomposites. RGO agglomerates and voids can be clearly seen when the RGO loading is increased from 0.5 to 2.0 wt %.

#### 3.3.2. Transmission Electron Microscope (TEM)

**Figure 13:**
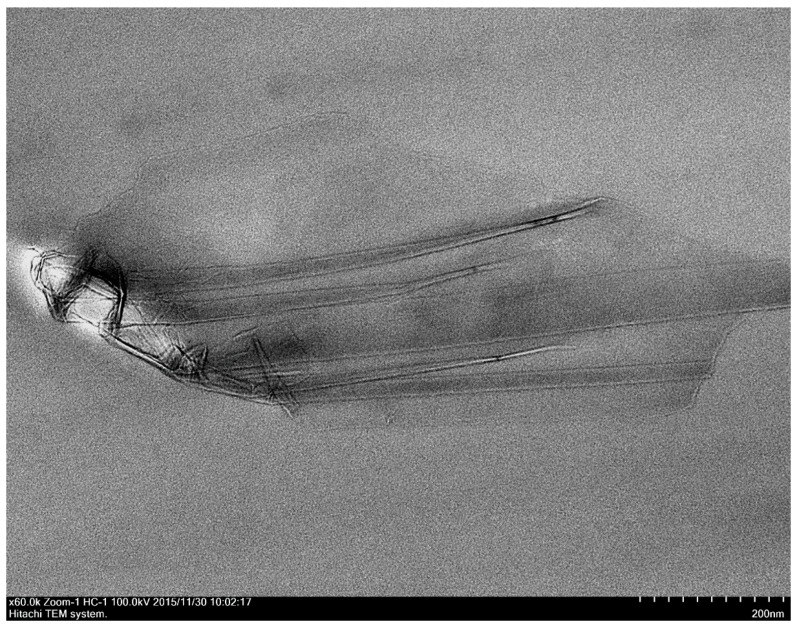
Cross section TEM image of RGO.

**Figure 14:**
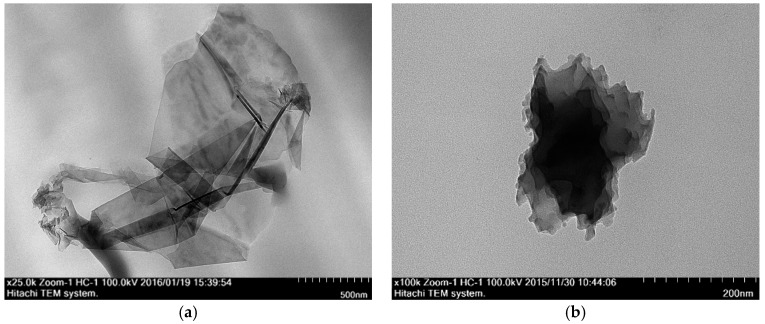
Cross section TEM images of nanocomposites prepared by in situ polymerization with different RGO loading. (**a**) 0.5 wt % RGO loading; (**b**) 2.0 wt % RGO loading.

**Figure 15:**
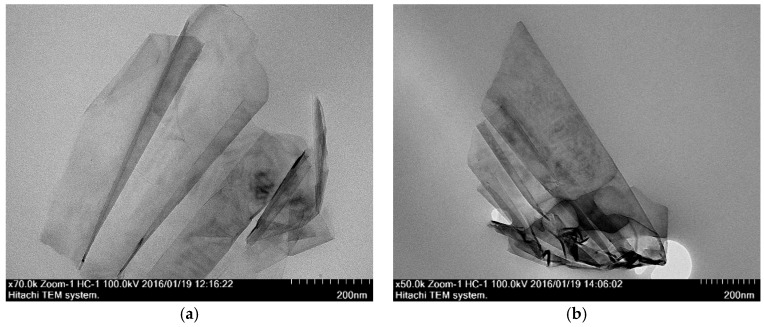
Cross section TEM images of nanocomposites prepared by solution blending with different RGO loading. (**a**) 0.5 wt % RGO loading; (**b**) 2.0 wt % RGO loading.

RGO is a smooth and thin layer of sheets stacked together as shown in Figure 13. The number of layers ranges from 2 to 4 by visual inspection, and the average length is found to be 738.5 ± 141.1 nm. In the present study, nanocomposites prepared by solution blending have a better RGO dispersion than those prepared by in situ polymerization. This can be confirmed from the cross-section TEM images of nanocomposites prepared by in situ polymerization and solution blending methods, shown in Figure 14 and Figure 15, respectively. Apparently, the nanocomposites prepared by in situ polymerization have more aggregate numbers of multi-layer RGO, which is densely agglomerated, as indicated by the darker image of multilayer RGO.

### 3.4. Solar Energy Conversion Efficiency

#### 3.4.1. Specific Heat Capacity

**Figure 16:**
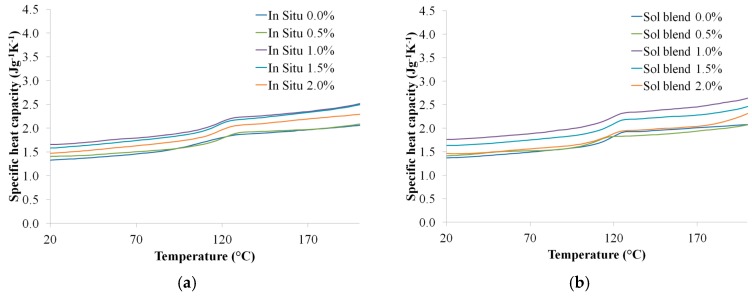
Specific heat capacity of the neat PMMA and RGO/PMMA nanocomposites prepared by (**a**) in situ polymerization and (**b**) solution blending.

The calculation results of specific heat capacities from the DSC are displayed in Figure 16. Generally, PMMA and its nanocomposites prepared by solution blending have higher heat capacities than those prepared using the in situ polymerization method.

#### 3.4.2. Solar Energy Conversion Efficiency

**Figure 17:**
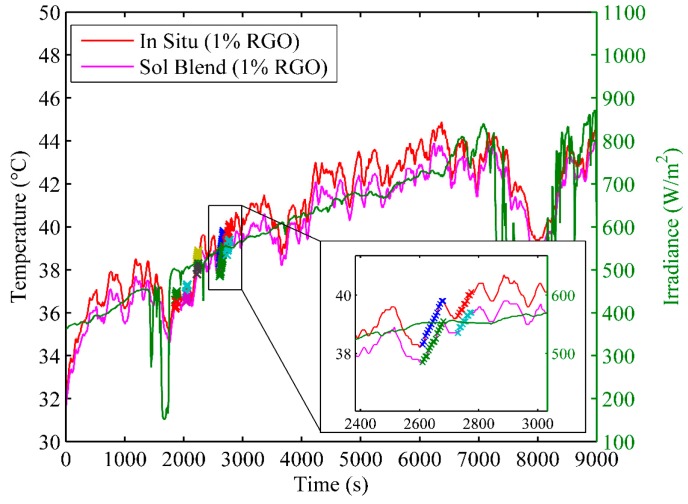
Sets of results that fulfill the required conditions.

The nanocomposites were thermally insulated at all surfaces except the top surface during the outdoor experiment. The thermal energies absorbed by the nanocomposites were calculated from the rise in temperature of the nanocomposites. Heat loss is assumed to be negligible. The temperature of the nanocomposites shows an increasing trend when taking the measurement under constant solar radiation. Figure 17 shows the daily temperature data which fulfilled the measurement conditions as follows:

(1) The starting temperature ranged from 30 to 40 °C;
(2) The solar irradiance ranged from 500 to 1000 W/m^2^;
(3) The temperatures of all nanocomposites increased for more than 20 s;
(4) The changes in solar irradiance were less than 1% during the period of time.

**Figure 18:**
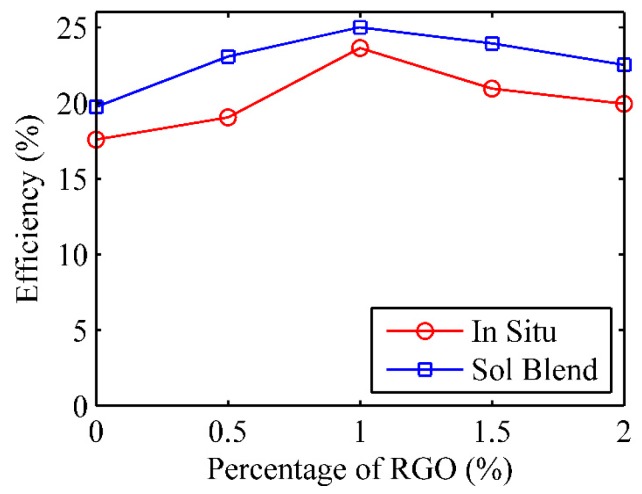
Solar energy conversion efficiency of the neat PMMA and its nanocomposites.

The results of solar energy conversion efficiencies of 10 neat PMMA and PMMA/RGO nanocomposites prepared by the two different methods are compared in Figure 18. The efficiencies in the graph are the average efficiencies of 25 results obtained from several days of outdoor experiment that met all required conditions. The energy conversion efficiency of the PMMA and its composite prepared by the solution blending method was found to be relatively higher. Besides, the maximum efficiency of about 25% can be achieved when 1% RGO was added to the PMMA. This may be due to the fact that the surface of neat PMMA without RGO is white in color. By adding RGO, the surface turns black, resulting in higher absorptivity and lower emissivity of solar heat. Consequently, the energy conversion efficiency is enhanced. Nevertheless, subsequently increasing the RGO to 2% did not improve the efficiency in the present study. This may be because both the absorptivity and emissivity were high in this case; hence, the surface emitted large amounts of thermal radiation in the form of radiative heat loss to the environment, and this decreased the efficiency.

**Figure 19:**
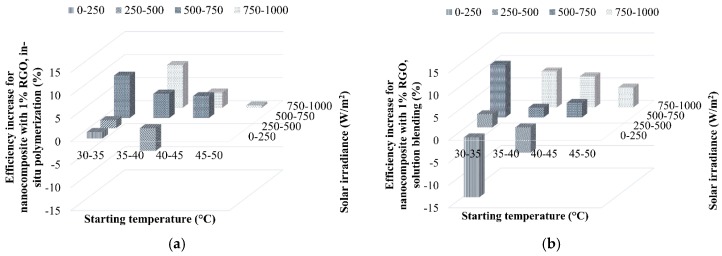
Increment of efficiencies with 1% RGO prepared by (**a**) in situ polymerization and (**b**) solution blending.

In order to investigate the effect of solar irradiance, the efficiency increment of nanocomposites mixed with 1% RGO is illustrated in Figure 19. The optimum temperature for the nanocomposites is found as a range of 30–45 °C. In addition, the nanocomposites may absorb the heat without significant heat loss when the solar irradiance is 500–1000 W/m^2^. As a result, the nanocomposites prepared by the solution blending method exhibit greater conversion capability than those prepared by in situ polymerization within the measurement domain.

It is worth noting that the nanocomposites prepared by in situ polymerization perform better under a low solar irradiance of 500–750 W/m^2^, whereas those by the solution blending method require a higher solar irradiance of between 750 and 1000 W/m^2^. Furthermore, when the solar irradiance is very low during cloudy periods, ranging from 0 to 500 W/m^2^, the efficiency of the nanocomposites is lower than that of the neat PMMA without RGO loading. This is because the negative contribution of the heat loss to the environment through the top surface of the nanocomposites is more significant in this case due to the low solar irradiance. The heat loss, as the dominant effect, would be increased by the darker surface of the nanocomposites with RGO owing to the higher emissivity. Therefore, the efficiency decreased.

## 4. Conclusions

The results from spectroscopic studies using FTIR, Raman spectroscopy and XPS confirmed that the GO was successfully reduced to RGO. Ductile behavior can be seen in the stress-strain curves for the neat PMMA prepared by the in situ polymerization method. However, all other samples displayed brittle fracture behavior. Better dispersion of RGO in the PMMA matrix of the solution blended nanocomposites can be confirmed from the Scanning Electron Microscope (SEM) and Transmission Electron Microscope (TEM) results. The optimum concentration of RGO/PMMA nanocomposites is found as 1.0 wt % that yields the maximal solar energy conversion efficiency of about 25%.

## Figures and Tables

**Table 1:** Mechanical properties for neat PMMA and its nanocomposites.

Method	RGO loading (wt %)	Young’s modulus (MPa)	Tensile strength (MPa)	Elongation at break (%)
In situ	0.0	362.28 ± 9.79	47.73 ± 1.75	29.96 ± 2.77
	0.5	428.51 ± 32.32	44.10 ± 3.02	13.50 ± 1.13
	2.0	288.72 ± 48.96	29.68 ± 1.25	13.95 ± 1.76
Solution Blending	0.0	330.47 ± 26.00	35.89 ± 1.88	15.10 ± 2.61
	0.5	463.02 ± 47.66	36.64 ± 1.18	10.02 ± 1.10
	2.0	470.72 ± 33.34	25.51 ± 6.01	6.94 ± 1.13
